# Reaffirming the republican dilemma: Replies to critics

**DOI:** 10.1177/14748851251378929

**Published:** 2025-09-24

**Authors:** Lars JK Moen

**Affiliations:** 1 Department of Philosophy, 27258 University of Vienna, Vienna, Austria

**Keywords:** Liberalism, pure negative freedom, republican freedom, republicanism

## Abstract

In *The Republican Dilemma*, I argue that contemporary republicans fail in their attempts to distinguish their theories from a liberalism fundamentally concerned with individuals’ pure negative freedom. I also show how achieving such a break from liberalism takes republicanism towards a political ideal unsuited for modern pluralistic societies. In this paper, I reply to four commentators and their challenges to core arguments in my book.

## Introduction

I appreciate this opportunity to respond to the criticisms made by the four participants in this symposium on my book, *The Republican Dilemma: Promoting Freedom in a Modern Society*. In the book, I argue that defenders of the ideal of republican freedom must either sacrifice a concern with pluralism or accept the ‘equivalent-judgments thesis’, which says that republicans’ judgments about how to measure and promote freedom will not differ significantly from those of theorists holding a purely negative conception of freedom that republicans claim to oppose. Here I will show why I think my critics fail in their attempts to challenge this central argument in the book. I address my critics’ papers separately in the order in which they appear in the symposium.

## Freedom among equals

Devon Cass introduces the case of a slave with a benevolent master, which is frequently used by republicans to show how their account of freedom is significantly different from negative freedom as the absence of interference or prevention. The slave avoids the master's interference by acting deferentially towards her, thus maintaining her goodwill towards him. The master, in Cass's example, does not restrict the slave's ability to choose between eating apples or oranges. Cass says the slave has pure negative freedom in this choice, but he is unfree in the republican sense because the master has the power to interfere.

To be precise, pure negative freedom is not about choices but actions, as it says you are free to do some action, *x*, as long as no one prevents you from doing *x*. And you are unfree to do *x* if and only if someone prevents you from doing *x*.^1^ Prevention here means making it physically impossible for you to do *x*. And it does not matter why you are prevented or whether the prevention is intentional, justified, or permissible. This means the slave in Cass's example is free to eat apples if no one prevents him from doing so, and free to eat oranges if no one prevents him from doing so. Republican freedom as non-domination, on the other hand, is a status you enjoy under institutions denying others the power to interfere with you without concern for your interests. Being unfree is to be in a vulnerable position in which others can interfere with you as it pleases them. The slave finds himself in such a position in relation to his master and is therefore considered unfree regardless of how the master happens to treat him.

But as Cass notes, the slave example does not show that republicans and pure-negative-freedom theorists will make different judgments of how free someone or some society is, or how freedom should be promoted in a society (pp. 48–56).^2^ Crucially, in virtue of her power, the master makes the slave unfree to do various things that would lead her to prevent the slave from eating apples or oranges. This captures the republican concern with dominated people having to act so as to maintain the goodwill of their superiors. The master thus restricts the number of sets of conjunctively exercisable opportunities the slave has that include opportunities to eat apples and oranges. The master thus reduces the slave's overall pure negative liberty. Also the promotion of pure negative freedom will therefore involve denying the master the power she has over her slave.

But Cass suggests judgments based on these different conceptions of freedom will differ in a type of case where the master is ‘ultra-kindly’, as she will not interfere with the slave regardless of how he acts towards her. This slave, we may imagine, will suffer no punishment if he decides not to work, he can use his master's property as it pleases him, and he can run, or walk, away because the master will not stop him. This is a peculiar kind of master–slave relationship, but as Cass notes, I say in the book that such a slave would still be prevented, even if not by the master, from doing certain things, including actions associated with citizenship (p. 55). But it seems safe to say this slave will have more pure negative freedom than a slave with a more restrictive master.

Cass says this case ‘seems to put substantial pressure on the equivalent-judgments thesis’. For the republican, he says, even this slave will be unfree in all of his choices because the master can interfere with impunity. It does not matter how likely the master is to interfere. To further illuminate this point, Cass introduces a case where dominated individuals are denied no opportunities in virtue of being dominated. In ‘The Society of Benevolent Patriarchs’, women have the same opportunities as men. Every man is ‘ultra-benevolent and would never interfere’. But ‘by the standards of the society, husbands are regarded as entitled to interfere with their wives' choices with impunity’. So, no man would ever interfere, but were he to do so, that would not be frowned upon. Women are inferior in this society, Cass says, even though they are never denied any opportunities enjoyed by men.

Here we have a society where men appear to be strictly compliant with a norm of not restricting what their wives can do. But peculiarly, it is widely accepted that every man is entitled to do so. Cass seems to be right when he says ‘there would be no basis in pure negative liberty to introduce law that would protect women—and indeed such law would reduce the pure negative liberty of men’. And we might add that insofar as women are taxpayers, their pure negative liberty would also be reduced, since tax money would presumably have to be spent on the making and enforcement of these laws, and taxpayers could have spent this money on other things. Cass's view of a republican is someone who would insist on costly and restrictive legal institutions even in this unrealistic case where such institutions would only deny individuals opportunities without giving them any in return.

While this understanding of republican freedom does seem to challenge the equivalent-judgments thesis, I do not think republicans can coherently take this view. A significant part of the book, which Cass does not mention, is the discussion of the republican concern with people's common interests. This concern imposes a constraint on how much institutional protection is justifiable within a republican framework, and therefore on how restrictive the institutions said to constitute republican freedom can be. I show in the book how the account of common interests we find in Pettit's republicanism is very similar to the one we find in Rawls's political liberalism (ch. 4). The important point in response to Cass's comment is that a concern for common interests, thus understood, will constrain the extent to which institutions can justifiably deny people opportunities in order to protect them against each other's interference. That is because such institutions are themselves necessarily interfering, and they will not be compatible with common interests in cases, very unlikely to materialize in large modern societies, where they would only deny people opportunities without giving them any in return.

So, while republicans might falsify the equivalent-judgments thesis by taking the line Cass suggests, they must then also adjust their account of common interests. And such an adjustment, I argue, will not be friendly to the liberalism and pluralism most contemporary republicans seem to endorse.

## Freedom at different levels

Victoria Costa observes that while pure-negative-freedom theorists consider any form of prevention a source of unfreedom, republicans think only morally objectionable interference makes individuals unfree. She therefore agrees with me that republican freedom is a moralized conception of freedom (ch. 2; see also Moen, 2023). But she takes this observation to challenge the equivalent-judgments thesis I defend. By considering only objectionable interference a source of unfreedom, she argues, we cannot make the same freedom judgments as someone who takes any act of interference to make us unfree.

But it does not follow from this observation that the moralizer and the pure-negative-freedom theorist must make different judgments of how free people are or how freedom can be promoted in a society, which is what the equivalent-judgments thesis is about. To see how these judgments can be the same despite the difference Costa points out, we should note that the two conceptions are formulated at different levels (p. 34). Pure negative freedom is formulated at a basic level prior to any normative theorizing about how individual ought to treat each other or how institutions ought to function. A moralized conception of freedom, like republican freedom, on the other hand, is defined at a higher level, as it is based on a normative theory telling us what kind of interference is justified, or unobjectionable, and what kind is not.

Costa effectively says we cannot get equivalent judgments because the two conceptions of freedom are defined at different levels. But different levels do not mean different judgments about how institutions can promote freedom. To compare these judgments, we first note that republican freedom is a status one enjoys under a particular institutional arrangement. We can then consider what ensuring people this status means in terms of their pure negative freedom. In a large, modern society, it will no doubt require institutional constraints that deny them certain liberties. But those constraints may nonetheless be justified in terms of pure negative liberty insofar as it also ensures people opportunities they otherwise would have been denied. Institutions ensuring, or constituting, people's status of republican freedom may therefore promote their overall pure negative freedom—and the equivalent-judgments thesis says that they do.

Costa's second point of criticism is that my discussion of the similarities between Rawls's liberalism and Pettit's republicanism cannot support the equivalent-judgments thesis because Rawls is no pure-negative-freedom theorist. Rawls takes only some humanly imposed obstacles to make people unfree, and, like Pettit, he is particularly concerned with the basic liberties and individuals’ capacity to exercise them. Costa does not deny considerable similarities between Rawlsian liberalism and contemporary neo-republicanism, but she does deny that these similarities can support the equivalent-judgments thesis because neither position defends pure negative liberty.

But with a clear view of the different levels at which republican freedom and pure negative freedom are defined, we see that support for the promotion of the more basic pure negative conception of freedom need not be explicit. Neither Rawls nor Pettit is a pure-negative-freedom theorist in the sense of defining freedom as pure negative freedom and explicitly defending its value. But my comparison of their theories in the book is meant to reveal a shared fundamental concern with individuals’ pure negative liberty. This is particularly evident in their similar accounts of common interests (ch. 4) and their shared commitment to pluralism (ch. 6). While their concern with pure negative liberty is mostly implicit—and only occasionally explicit (e.g. pp. 83–84)—it is a mistake to say they do not value pure negative freedom just because they define freedom at a higher level on the basis of what they consider justifiable or permissible in their theories, since pure negative freedom can—and does—have a fundamental place in these theories.

## Institutions and freedom judgments

Sean Ingham devotes most of his attention to showing why he thinks the equivalent-judgments thesis, or at least one version of it, is false. But he first suggests it would be unproblematic for republicans, even if true, if it only means convergence ‘on certain institutional prescriptions and normative judgments about citizens' responsibilities’.

Republicans attempting to show how republican freedom has more attractive implications in terms of institutional requirements would probably disagree with this view. Pettit suggests a concern for negative freedom could undermine efforts to establish institutional protection since it implies that people in an inferior position can simply liberate themselves by acting deferentially towards more powerful individuals to gain their goodwill and thereby avoid their interference. In support of the equivalent-judgments thesis, I address these concerns, and I show how Pettit's ‘liberation by ingratiation’ argument rests on a misunderstanding of pure negative freedom (pp. 52–54).

Another way equivalent judgments would be a problem for republicans is implied by pure negative freedom being a more basic freedom concept, in the way I describe above in response to Costa above. Equivalent judgments would then mean pure negative freedom can be used to justify the institutions republicans take to constitute freedom. Pure negative freedom can therefore play a role in justifying the institutions republicans support, while republican freedom cannot because it is itself defined in terms of the justified institutions.

But Ingham focuses primarily on falsifying the equivalent-judgments thesis. It is false, he says, at least if we understand it to be about ‘judgments about how two individuals compare with respect to their freedom overall’. This thesis is stronger, he says, than one concerning ‘normative judgments about how institutions ought to be designed, which policies are best, or the degree to which citizens must devote themselves to public affairs, for example’. He does not deny that this weaker thesis about institutions is correct.

But this way of distinguishing between two versions of the thesis cannot work because there is no getting away from republican freedom being about institutions. A distinct feature of republican freedom is that it is brought into existence by institutions. We therefore cannot start to talk about republican freedom, let alone how to measure it, before we have worked out what institutions it requires. To consider an individual's republican freedom, we must look at what institutions are in place to protect the individual from a certain kind of interference. A republican therefore cannot judge ‘how two individuals compare with respect to their freedom overall’ without also making ‘normative judgments about how institutions ought to be designed’. The version of the equivalent-judgments thesis concerning institutional requirements that Ingham makes no attempt to reject is therefore the one we have to take.

The attempt to ignore institutions causes problems for Ingham in his exploration of isolated cases of relationships between individuals. Because of the institutional nature of republican freedom, we cannot consider the freedom in these relationships without relying on considerations of what institutions should be in place in the society. Ingham first considers the case of a slave with ‘a robustly benevolent master, one whose benevolence is not predicated on the slave's obsequiousness or any other behaviour of the slave's’. This seems to be essentially the same kind of case as the one Cass introduces in his contribution to this symposium, which I respond to above.

But Ingham develops a different case to show how his preferred account of republican freedom leads him to judge a person to be unfree despite no one reducing their pure negative freedom. Here the dominator is a husband with the power to constrain his wife's opportunities to seek employment outside the home. But as it happens, the husband is ‘robustly benevolent’ and will not interfere with his wife no matter what she decides. The wife also knows this.

The source of unfreedom Ingham sees, and he claims pure-negative-freedom theorists fail to see, is that it is not common knowledge in the society that the husband is a benevolent, non-interfering type. In particular, this affects a potential employer who considers hiring this woman. But the wife is still constrained—her pure negative freedom is reduced—by someone. It seems the dominator is not the one making the woman unfree in this case, but she is still made unfree by someone. And someone makes another unfree by preventing them from doing something regardless of why they do so—that is, indeed, what makes the conception of liberty purely negative. Here, then, we seem to have a structural issue that explains why someone is made unfree to do something in the pure negative sense.

Ingham also notices this and responds by adjusting the case so that the woman is in no way restricted. It is not common knowledge that the husband is benevolent and non-interfering, but the employer will nonetheless hire the woman if she applies. But the lack of common knowledge about the husband's benevolence is still practically significant, Ingham claims. When the employer meets the couple at a social gathering, he feels unsure about the husband's attitude towards his wife seeking employment, and he therefore refrains from bringing up the job in his presence. Had he been assured of the husband's benevolence, he would have brought it up. The lack of common knowledge about his benevolence is therefore practically consequential even though it leads no one to prevent anyone else from doing something.

It is not quite clear who Ingham takes to be unfree in this case. I assume it is the woman, but it is the employer's behaviour that is affected by the lack of common knowledge about the husband's benevolence. Or perhaps everyone is unfree because of the absence of freedom-constituting institutions. At any rate, a republican's judgments about the freedom in this case must be based on considerations of what institutions ought to be in place. For Ingham, these are institutions that would ensure common knowledge of the husband's benevolence.

Such considerations involve an analysis of whether costly and restrictive institutions are justified when they will not actually provide anyone any opportunities. In Ingham's case, they will not plausibly be justified only to make one individual feel he can speak freely about a particular subject in the presence of one particular individual. It seems more apt to consider whether institutions ought to be in place to actively restrict husbands’ opportunities to prevent their wives from seeking employment outside of the home, even though no husband would do so in the absence of such institutional constraints. And remember that wives also know this about their husbands. Everyone else in the society can therefore also observe that wives seek employment outside of the home unconstrained by their husbands.

This brings us back to the conclusion I reached in my response to Cass. Introducing costly institutional protection in such cases will only deny people opportunities—including women forced to contribute to the maintenance of these institutions. Republicans would need a restrictive account of common interests to support such institutions, one that does not fit with the liberal concern with pluralism that we find in most contemporary republican writings. Ingham tries to ignore this concern by leaving institutional requirements out of the picture, but he cannot do so when considering the requirements of a conception of freedom constituted by institutions.

## Moralization, law, and pluralism

Frank Lovett starts by objecting to my way of arguing that republican freedom is a moralized conception of freedom. He says he agrees with my understanding of what makes a conception of freedom moralized and does not attempt to deny that republican freedom is moralized. But he objects to what he takes to be my view that any possible definition of freedom that distinguishes between one kind of prevention that makes you unfree and one kind that does not is necessarily moralized. Here I doubt that Lovett means I take any *possible* way of making such a distinction to be based on moral evaluation. We can say that only preventions occurring on days starting with T make you unfree, and then give the reason that the letter T is aesthetically unappealing. The distinction between one kind of prevention that makes you unfree and one that does not is then not based on moral evaluation. What Lovett presumably means is that I take something like any plausible way of making the distinction to be based on moral evaluation of what is permissible or justifiable.

Lovett introduces two cases to show why he thinks a plausible way of making the distinction need not be based on moral evaluation. We may think children should be prevented from voting because their capacities for political reasoning are not yet fully developed. Determining whether a particular child possesses the relevant reasoning capacities, Lovett says, would require ‘some sort of evaluative judgment as to her political reasoning capacities’. But to effectively enforce a rule denying children the opportunity to vote, Lovett thinks we should avoid such evaluation and simply say ‘persons under the age of 18 shall not vote’. We then just check people's dates of birth to decide who can vote and not. Similarly, Lovett adds, we need no normative evaluation to say whether a case of interference has been supported by a majority of voters. Whether the interference has such approval, he says, ‘is presumably an observable, descriptive fact that ordinarily does not require evaluative judgment’. So, we need not rely on moral evaluation when we say only people aged 18 or over are made unfree when denied the opportunity to vote or that only interference not supported by a majority is a source of unfreedom. The judgment about freedom in these cases, Lovett says, concerns ‘descriptive facts alone’. The reasons for making the distinction between freedom and unfreedom are normative, but that, Lovett says, does not make the conception of freedom moralized.

Lovett does not mention that Pettit (2006: 279–280) also makes this attempt to show that his conception of republican freedom is not moralized. Nor does he mention my response to Pettit in the book (pp. 28–30). Pettit thinks only interference not under popular control is a source of unfreedom, and he points out that whether an act of interference is controlled in this sense is a factual matter. But this is not what matters for determining whether a conception of freedom is moralized. What matters is the reasons for distinguishing unfreedom-causing interference from other interference—the reasons Lovett wrongly claims do not matter. These are the reasons we appeal to when we say interference that makes people unfree is unjustified, while other interference is justified and therefore compatible with people's freedom. In Lovett's first case, we justify denying people under 18 the opportunity to vote because of the ‘good normative reasons’ that Lovett give concerning children's undeveloped reasoning capacities. Similarly, the view that popularly controlled interference or interference supported by a majority is compatible with freedom must also be based on reasons for thinking such interference is justified. Once the justification is formulated, and we use it to distinguish freedom from unfreedom, we can perhaps agree, as a matter of fact, whether a particular act of interference fits into one or the other category. But the distinction is nonetheless based on normative judgments about what makes the kind of interference we consider compatible with freedom justified. That is what makes the conception of freedom moralized.

But Lovett thinks that if the distinction between prevention that makes us unfree and prevention that does not must be based on moral reasons, then pure negative freedom is also moralized. After all, it says only the prevention by another agent makes us unfree. Natural obstacles, for example, do not. But on the understanding of moralization that Lovett says he and I share, this is incorrect. On this view, going back at least to Cohen's (1979) critique of Nozick, the relevant distinction is between two kinds of prevention by an agent, not agential prevention and non-agential prevention. On a moralized view, we say that one kind of agential prevention is a source of unfreedom while another kind of agential prevention is not. On the non-moralized pure negative view, we say that all agential prevention is a source of unfreedom. Whether the prevention is justified or not is a separate issue. We therefore say that A makes B unfree to do *x* by preventing B from doing *x*. And A makes B unfree to do *x* regardless of how justified we may think the prevention is.

We can, of course, also consider why only *agential* prevention makes us unfree to do some action, and not also other things that can make it impossible for us to perform the action. But such consideration will not involve any judgment about whether a prevention is justified or not. It instead concerns whether the distinction between freedom and unfreedom should be the same as that between ability and inability, and I will not enter into that debate here.

Lovett then turns to my discussion of the scope and robustness dimensions of freedom (chs. 2–3). Scope refers to the extent to which agential physical prevention is considered a source of unfreedom. Pure negative freedom has maximal scope because it treats all such prevention as a source of unfreedom. Republican freedom has a lesser scope because it identifies a kind of agential prevention that is not a source of unfreedom. Robustness concerns the extent to which freedom requires not only that no one prevents you from performing some action but that you are institutionally protected in such a way that you can perform the action in a range of possible worlds defined in terms of others’ preferences and their inclination to interfere with you. Pure negative freedom has no robustness since it simply says you are free to do *x* as long as no one prevents you from doing *x*. Republican freedom has greater robustness because it requires not only that no one stops you from doing *x*, but also that they would not do so regardless of how they may be disposed towards you. Republican freedom thus requires non-interference not just in the actual world but also in a range of other possible worlds.

I argue that there is a trade-off between scope and robustness in the sense that an increase in robustness must come with a corresponding decrease in scope. To protect people's opportunities to do certain things, they must be denied opportunities to deny each other those opportunities. So, not all opportunities can be protected. If freedom then requires robust protection, then there will be certain things people cannot be free to do, and preventing them from doing those things will not make them unfree. Any increase in robustness thus means a reduction in scope. Republican freedom gains robustness at the expense of scope as it picks out a certain kind of interference that does not make us unfree (ch. 3). Pure negative freedom, on the other hand, demands no robustness and can therefore treat all kinds of agential prevention as a source of unfreedom.

Lovett says this is just one possible way to define republican freedom and suggests republicans are not restricted by the scope–robustness trade-off. But when exploring alternative ways, Lovett sets aside scope and robustness and instead focuses on what he calls ‘domination’ and ‘frustration’ in a choice before and after a law has been introduced. These concepts are significantly different from what I call scope and robustness, and it is unclear how they might be useful in a discussion of the two dimensions I introduce. Lovett explains that someone dominates his choice ‘to the extent that she has an uncontrolled ability to frustrate one of the options in my choice set’. And she frustrates his choice ‘if she actively and intentionally blocks, burdens, or undermines one of the options in my choice set’. With this terminology, Lovett ignores my focus on scope as concerning all agential prevention, and not just intentional prevention. My analysis is also insensitive to whether a law has been introduced or not. Lovett thereby also disables himself from fitting pure negative freedom into his picture, though he suggests a vague, and seemingly mistaken, way of measuring pure negative freedom by counting how many non-frustrated and non-dominated choices a person has.

Lovett then says my view of a necessary scope–robustness trade-off means republican freedom is only about the absence of domination. But republicans, he says, can also account for what he calls frustration as a separate, albeit less weighty, concern in their measurement of freedom. He suggests ‘[t]he law prohibiting attacks does not reduce my freedom to attack others because I was not free to attack others before the law anyway’. Here Lovett seems to think law enforcement denying him the opportunity to attack others is non-frustrating because this action would be impermissible. ‘Frustration’ is then a moralized notion and does nothing to deny the fact that protecting others against his attacks involves physical prevention. He also suggests we can compare individuals’ freedom by first considering their non-domination and then their non-frustration only if the two are tied in terms of non-domination. But either way, by taking legal constraints to ensure non-domination and therefore freedom, republicans will regard some form of agential prevention not as a source of unfreedom, whether the prevention is classified as ‘frustration’ or not. They thus reduce the scope of freedom for the sake of robustness.

Finally, Lovett objects to my view that an account of republicanism that avoids the equivalent-judgments thesis must require that citizens commit to a republican way of life characterized by active vigilance of political powerholders. And such a view, I argue, conflicts with a respect for the pluralism of modern society (ch. 6). In response, Lovett introduces his own favoured ‘moderate’ understanding of republicanism, which does not require such a politically active citizenry and is therefore compatible with pluralism. This seems right. I also say repeatedly in the book that such a moderate account for republicanism is compatible with pluralism. But moderate republicans must then accept the equivalent-judgments thesis. Lovett makes no attempt to deny that conclusion.

## Notes

1 This is Carter (1999) and Steiner's (1994) bivalence account of pure negative freedom, according to which not being unfree to do *x* means one is free to do *x*. Kramer (2003) defends a trivalent account according to which one is neither free nor unfree to do *x* when one is unable to do *x* but not prevented by another agent from doing *x*.

2 Parenthetical page and chapter numbers refer to pages and chapters in my book (Moen, 2024).
